# Multivariate functional neuroimaging analyses reveal that strength-dependent face expectations are represented in higher-level face-identity areas

**DOI:** 10.1038/s42003-023-04508-8

**Published:** 2023-02-01

**Authors:** Helen Blank, Arjen Alink, Christian Büchel

**Affiliations:** Department of Systems Neuroscience, University Medical Center Hamburg-Eppendorf, 20246 Hamburg, Germany

**Keywords:** Perception, Sensory processing

## Abstract

Perception is an active inference in which prior expectations are combined with sensory input. It is still unclear how the strength of prior expectations is represented in the human brain. The strength, or precision, of a prior could be represented with its content, potentially in higher-level sensory areas. We used multivariate analyses of functional resonance imaging data to test whether expectation strength is represented together with the expected face in high-level face-sensitive regions. Participants were trained to associate images of scenes with subsequently presented images of different faces. Each scene predicted three faces, each with either low, intermediate, or high probability. We found that anticipation enhances the similarity of response patterns in the face-sensitive anterior temporal lobe to response patterns specifically associated with the image of the expected face. In contrast, during face presentation, activity increased for unexpected faces in a typical prediction error network, containing areas such as the caudate and the insula. Our findings show that strength-dependent face expectations are represented in higher-level face-identity areas, supporting hierarchical theories of predictive processing according to which higher-level sensory regions represent weighted priors.

## Abstract (web summary)

A multivariate fMRI analysis of participants trained to associate scene images with subsequent face images suggests that face expectations are represented in higher-level face-identity areas.

## Introduction

Perception is considered a process of hierarchical Bayesian inference in which prior expectations are combined with incoming sensory information^1–3^. Bayesian theories of neural processing, such as predictive coding^4^, propose that the strength of perceptual priors and sensory input determines the extent to which expectations influence the resulting ‘posterior percept’^5,6^. The strength of our predictions could be derived, for example, from tracking the stability of our environment or from the reliability with which particular cues precede a specific event. While less precise priors have a smaller impact on the representation and perception of sensory information, more precise priors have a stronger influence.

In previous studies, the presence and content of prior expectations were primarily inferred indirectly by examining brain responses to the presentation of expected and unexpected stimuli after some kind of prior had been induced^7,8^. Converging evidence shows that expected stimuli lead to reduced responses, whereas unexpected stimuli evoke increased responses^9,10^, a phenomenon also called expectation suppression^11–13^. In addition, the increased response to unexpected sensory input is interpreted as a signal of surprise, i.e., prediction errors due to violation of prior expectations^14,15^.

In a second approach, the presence and content of prior representations were examined directly by investigating induced pre-activations of expected stimuli (with functional magnetic resonance imaging (fMRI)^16,17^, magnetoencephalography (MEG)^18,19^, and intracranial cortical recordings^20^). For example, it has been shown that category cues (e.g., ‘face’ or ‘house’) modulate pre-stimulus fMRI activity in associated category-selective brain regions^16,21^. Hence, there is evidence that expected sensory input is pre-activated in corresponding sensory brain areas.

However, how item-specific priors and prior strength are represented in the brain is less clear^15,22^. Prior strength could be part of pre-activated sensory templates and thus be co-localized with the representation of prior content itself in sensory areas^2,8,18^. In this study, we tested how the brain represents and weights multiple expectations, specifically, whether expectation strength is represented in those regions that also represent the content of the prior, here expected face. Secondly, we tested how the representation of presented faces depends on the strength of the preceding prior.

The hierarchically organized network of face-sensitive regions from occipital over fusiform to anterior temporal areas of the human brain provides an attractive system for examining prediction principles^23–25^. It provides a pre-defined set of regions with different specific functional preferences for faces, in which lower-level regions such as the occipital face area (OFA) process facial features, the fusiform face area (FFA) processes facial features, as well as face identity, whereas a higher-level region in the anterior temporal lobe (aTL) processes view-invariant face identity^23,24,26–29^, specifically in the right hemisphere^30,31^. Neurophysiological evidence for predictive codes throughout the face-processing hierarchy was recently provided by high-level prediction errors in lower-level face regions of the macaque cortex^25^. This finding is in line with the suggestion that FFA represents the combination of face expectations and presented faces in form of prediction errors^7–9^. Higher-level face-identity expectations seem to be represented in the aTL. In macaques, identity representations in the face-sensitive aTL emerged at an earlier latency than in the middle face patch region of the temporal lobe, which is topographically homologous to the human FFA^32^. These findings suggest that face-identity representations emerge in face-sensitive regions in the aTL and that feedback from these anterior regions may be critical for context-dependent face representations in more posterior face areas.

**Fig. 1:**
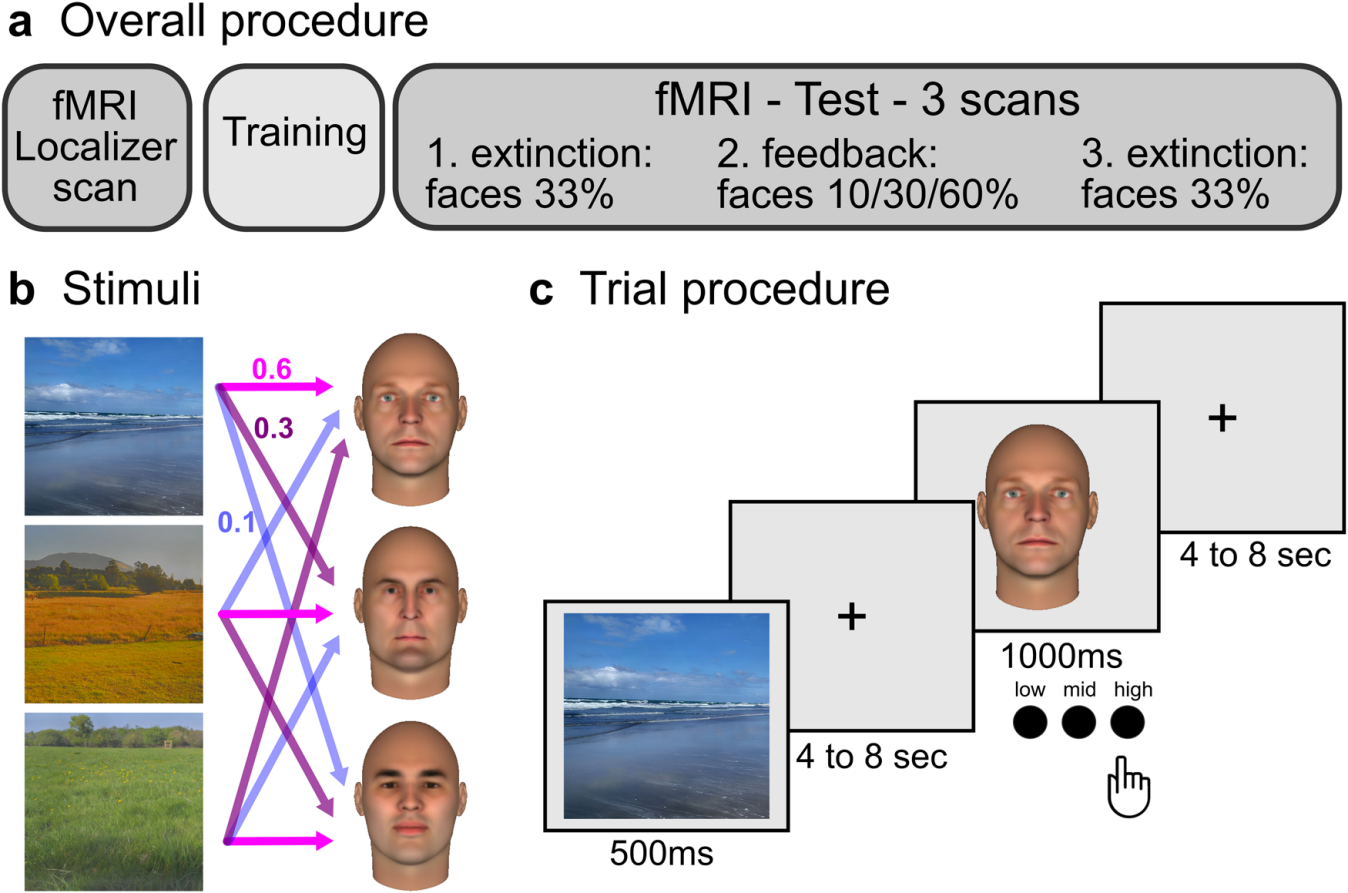
Procedure and stimuli. **a** The experiment started with a functional localizer in the scanner (30 min) in which participants saw images of three scenes and three faces (shown in **b**). This was followed by a training session outside the scanner (40 min). Finally, the main scanning session consisted of three test runs of which the first and the third were extinction runs, i.e., faces were presented with equal contingencies and no feedback about correct responses was provided. The middle run was a refresher training with feedback and faces were presented with learned contingencies (i.e., 0.1, 0.3, and 0.6, 60 min). **c** Trial procedure during the test. Scene cues were followed by face images showing three different identities. Participants had to indicate by button press how much they had expected the presented face, given the preceding scene cue (with low, mid, or high probability).

In the current study, we used functional resonance imaging (fMRI) in combination with multivariate methods to test whether the strength of face expectations can be detected alongside expected face images in face-sensitive regions (i.e., OFA, FFA, and aTL) of the human brain. Participants used scene cues to predict faces with different probabilities (Fig. 1). We found evidence that representations of expected faces were weighted according to their probability in the high-level face-sensitive aTL.

## Results

### Behavioral results

**Fig. 2:**
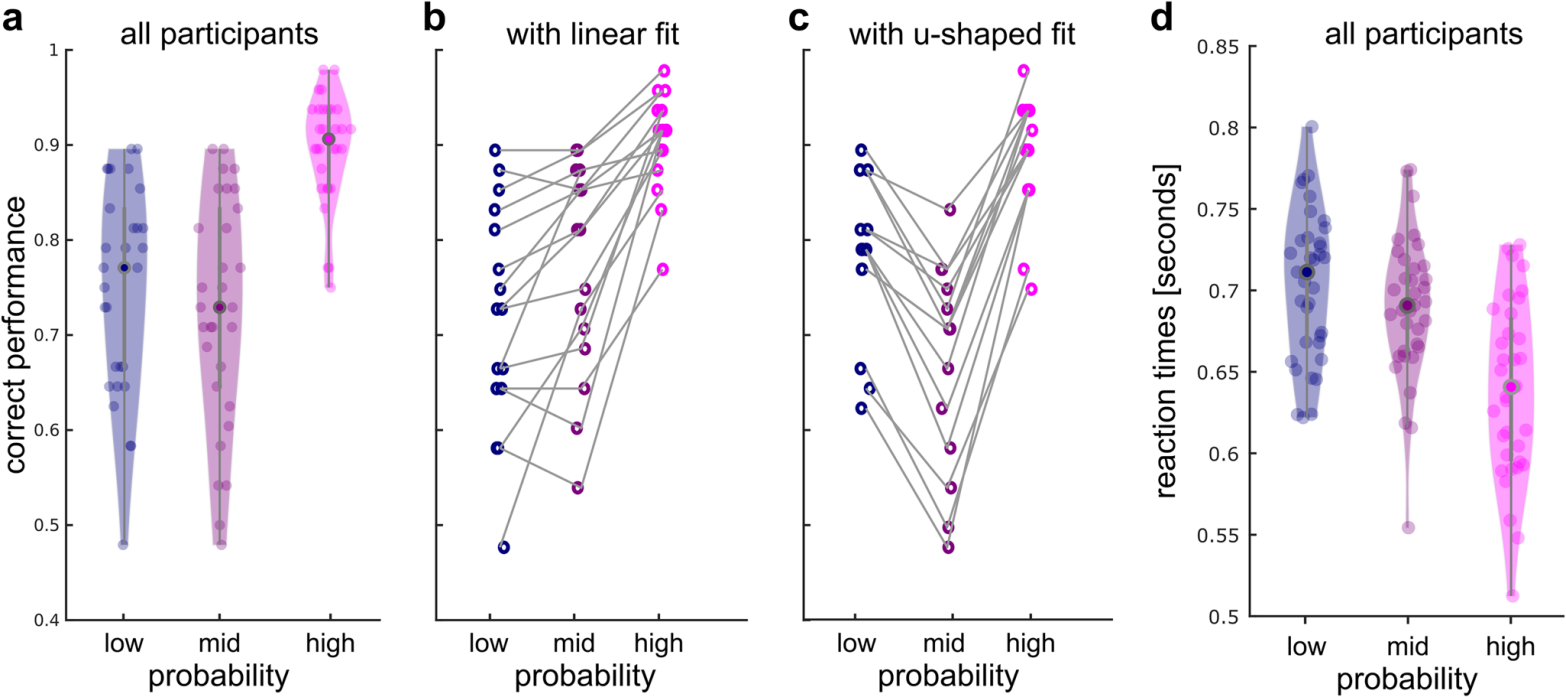
Behavioral results. **a** Correct performance in the face task. Participants learned to associate scene cues and face images with their corresponding probability. Participants could be split into two groups, based on whether their response profiles followed a linear (in **b**) or u-shaped fit (in **c**). This indicates that they might have used different strategies to anticipate the faces. **d** Reaction times in the face task. Participants responded faster to faces that were expected with high probability than to those expected with a low probability.

Participants associated the three scenes and faces with the corresponding low, intermediate, and high probabilities (mean correct responses averaged across test runs 1 and 3: 75.07% for low, 72.57% for intermediate, and 89.58% for highly expected faces, Fig. 2a). Inspection of individual correct performance revealed that participants differed in their response profiles: 16 participants showed a linear increase in percent correct with increasing face probability, whereas 14 participants showed a U-shape relationship and better performance for faces with low and high probability. These response profiles indicate that participants might have differed in the way they attributed strength to the scene priors, i.e., while the linear response profile indicates the intended attribution of low, intermediate, and high probability, the u-shape response indicates that participants might have used a different strategy such as anticipating the faces with low and high probability.

Participants responded faster to faces that were expected with high probability (mean reaction times in seconds across participants for low probability = 701 ms, mid probability = 688 ms, and high probability = 639 ms, Repeated Measures Single-Factor Analysis of Variance Test: *F*(2, 58) = 33.839, *p* < 0.001, n^2^ = 53.85, post-hoc *t*-tests for low vs mid probability: *t*(29) 1.9071, *p* = 0.0665; for low vs high probability: *t*(29) = 7.1668, *p* < 0.001, and for mid vs high probability: *t*(29) = 5.9147, *p* < 0.001).

In summary, behavioral results confirmed that participants used weighted prior expectations during face recognition.

### Univariate fMRI responses to presented faces depending on their expectation strength

**Fig. 3:**
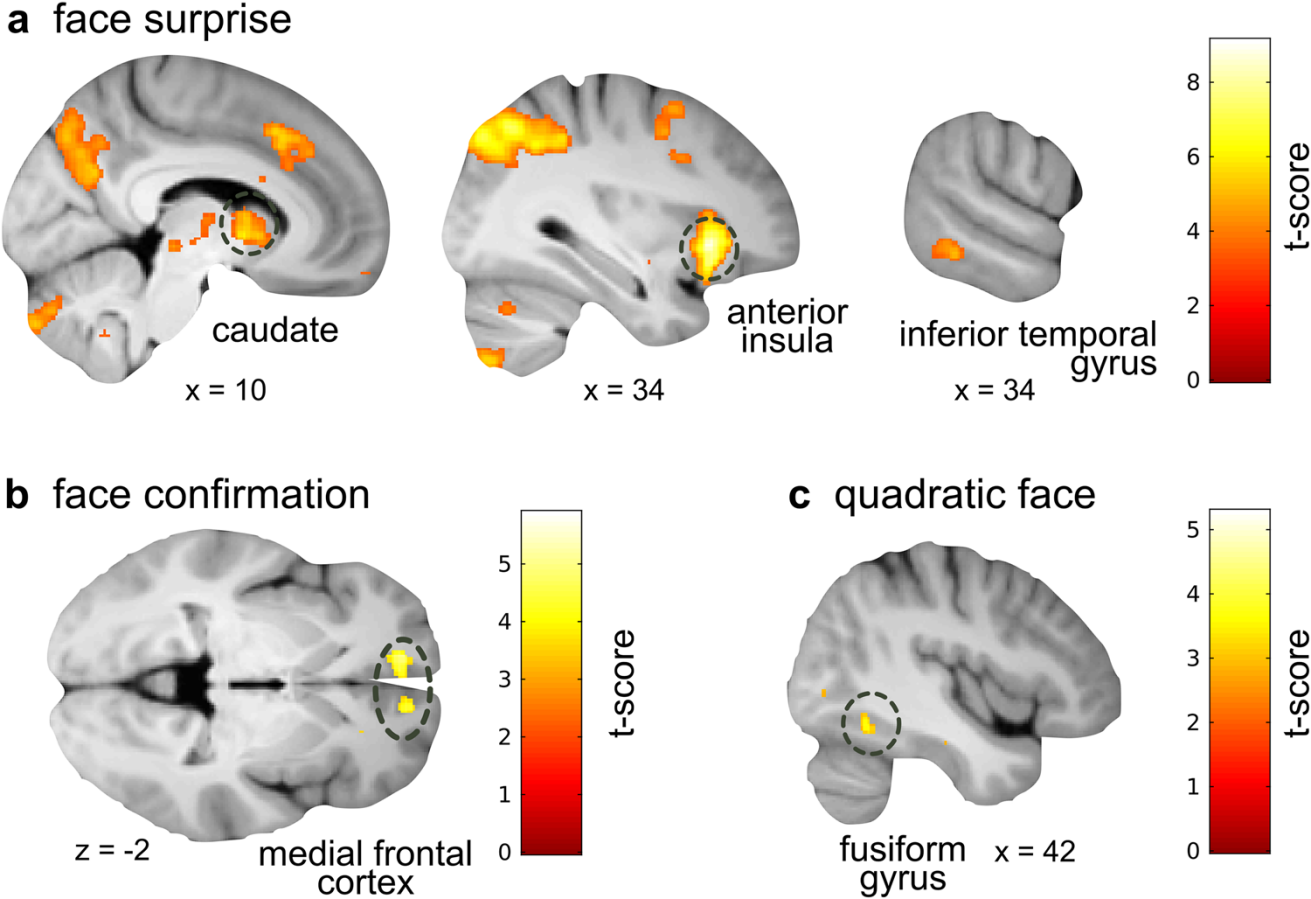
Univariate fMRI responses to presented face images depending on how much they were expected. **a** Activity in a typical network related to surprise (e.g., including the insula, caudate, inferior parietal lobe, and a cluster in the inferior temporal gyrus) increased with face surprise (negative linear parametric modulator, shown at *p* < 0.001, uncorrected). **b** Face confirmation, i.e., more expected faces, lead to stronger responses in the medial frontal cortex (positive linear parametric modulator). **c** U-shape response to faces reflecting increased activity for highly expected and unexpected faces was identified in the fusiform gyrus (quadratic parametric modulator).

Next, we turned to investigate whether our expectation manipulation influenced the univariate responses to presented face images depending on how much a presented face had been expected. Since each presented face had been either expected with low, mid, or high probability, we could test how the evoked univariate fMRI signal was modulated by the respective probability. Presentation of more unexpected faces elicited stronger responses in a typical network related to surprise^33,34^ containing activation of bilateral caudate, anterior insula, middle frontal gyrus, parietal lobe, precuneus, and superior frontal gyrus (negative linear parametric modulator testing for increased signal in response to presented faces that were less expected, Fig. 3a, Supplementary Table 2).

**Fig. 4:**
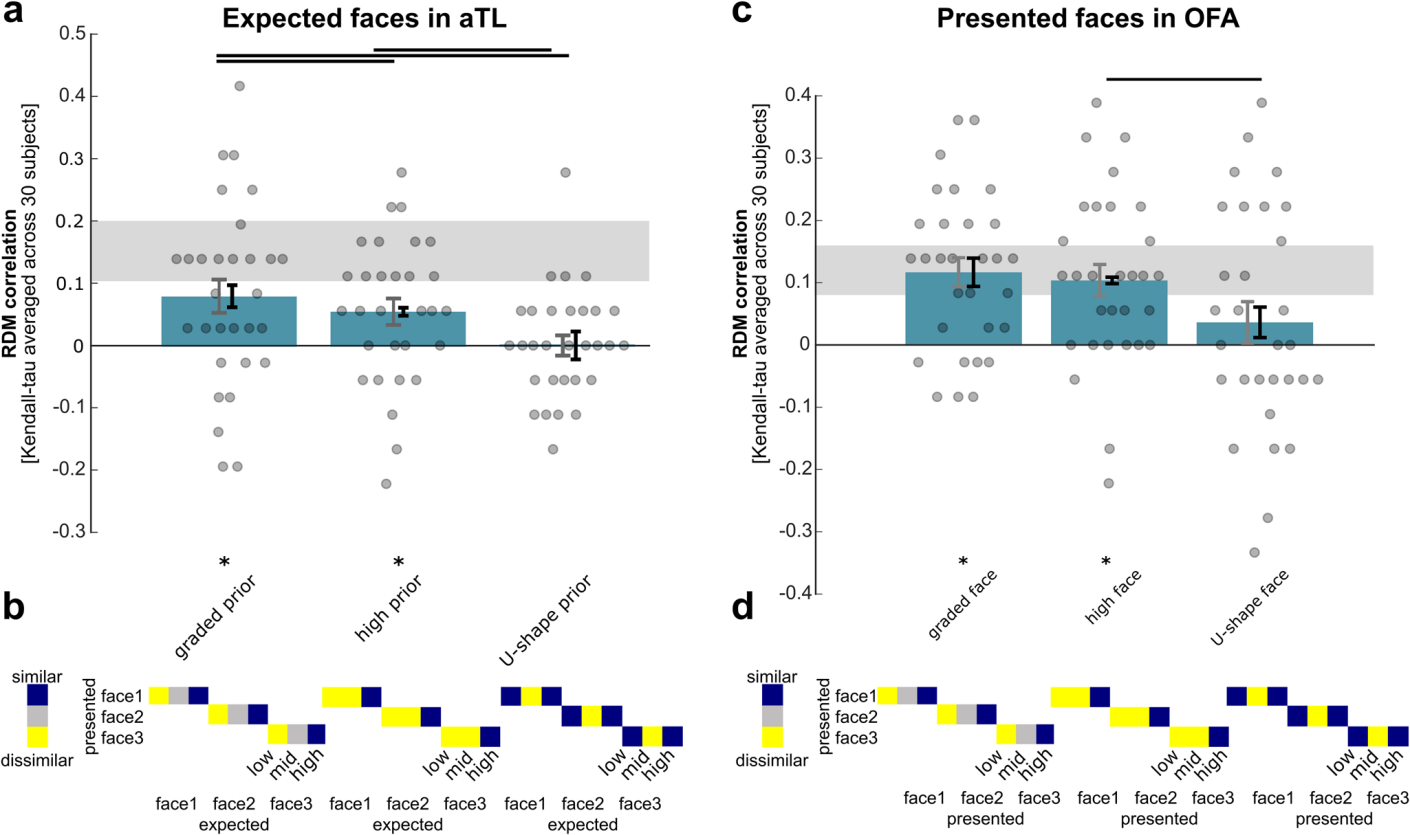
Multivariate fMRI results. **a** Representation of expected faces in the anterior temporal lobe face region (aTL). The correlation between the reference representational dissimilarity matrix (RDM) and the hypothesis RDMs was used to measure the performance of each hypothesis RDM. Gray error bars indicate the standard error of the mean, based on the across-subject variation, black error bars indicate the within-subject standard error of the mean^86^, and asterisks indicate significance with FDR control *p* < 0.05. The black horizontal lines indicate the pairwise comparisons for which the hypothesis RDMs perform significantly differently (FDR controlled *p* < 0.05). The gray horizontal bar shows the noise ceiling, i.e., the upper and lower bound estimates of the maximum performance any model could achieve given the level of noise in the data. **b** The hypothesis RDMs tested representations of graded expectations, only the high expectation, and a u-shape. The three hypothesis RDMs are presented in performance descending order. **c** Representation of presented faces in the occipital face area (OFA). **d** The hypothesis RDMs for testing the representation of presented faces, depending on how much they were expected. Again, the three hypothesis RDMs are presented in performance descending order.

In contrast, presentation of more expected faces activated the medial frontal cortex (positive linear parametric modulator testing for increased signal in response to presented faces that were more expected, Fig. 4b, x = −12, y = 50, z = −2, pFWE = 0.032 cluster-corrected, at *p* < 0.001 uncorrected, Fig. 3b).

Thirdly, motivated by the u-shaped response pattern in the behavioral responses, we tested for a u-shaped univariate response reflecting increased activity for highly expected and unexpected faces^35^. Indeed, activity in the right posterior FFA was higher for the highly expected and unexpected faces in comparison to the intermediate condition (quadratic parametric modulator, x = 42, y = −54, z = −14, k = 8, pFWE = 0.025, small volume corrected with the right posterior FFA ROI from the localizer identified on the group level, Fig. 3c).

### Multivariate representations of face expectations depending on their strength

To investigate how face expectations were weighted and represented we used multivariate representational similarity analysis (RSA^36,37^). A univariate approach was not suitable to measure the weighted representation of face expectations, as all three faces were simultaneously expected during a scene cue—but each with different expectation strength (e.g., scene 1 predicted face 1 with 0.1, face 2 with 0.3, and face 3 with 0.6 probability). Specifically, we tested whether the correlation between multivoxel representations of faces presented in the localizer and faces expected during the scene priors in the test runs depended on how strongly the respective face was expected (bottom of Fig. 4a).

Only in the high-level face-sensitive aTL region, RSA revealed representations of expected faces (Fig. 4a). Multivariate patterns of presented faces in the localizer correlated with graded face expectations (*r* = 0.0796, Z(29) = 2.6508, *p* = 0.004) and with the highly expected face (*r* = 0.0556, Z(29) = 2.4370, *p* = 0.0074) during scene presentation in the test runs. The corresponding hypothesis RDMs differed in their relatedness to the reference RDM and both differed also from the alternative RDM testing for a u-shaped expectation (black horizontal lines on top in Fig. 4a).

In contrast, in lower-level face-sensitive regions such as the OFA and the FFA, RSA did not reveal representations of expected faces (all *p* > 0.1 in the ROIs defined based on the group level). To not overlook representations of face expectations in lower-level face-sensitive regions, e.g., due to individual variability in the location of the FFA, we investigated activation patterns from individually localized posterior and anterior FFA regions. Also, in these individually localized FFA regions, we could not identify any multivariate representations of expected faces (all *p* > 0.1).

### Multivariate representations of presented faces depending on their strength

Finally, we applied this multivariate approach to test whether it would allow us to detect multivariate representations of presented faces depending on their strength that were not apparent in the univariate parametric approach (Fig. 3). Across the face-sensitive regions from OFA, over posterior and anterior FFA to the aTL, we only identified expectation-dependent multivariate representations of presented face images in the OFA (Fig. 4b). Multivariate patterns of presented faces in the localizer increased in similarity with graded face presentations (*r* = 0.1167, Z(29) = 3.8112, *p* < 0.001) and were more similar to the presented face that was highly expected than to those with low and intermediate probability (*r* = 0.1037, Z(29) = 3.2884, *p* < 0.001) in the test runs. The two corresponding hypothesis RDMs did not differ in their relatedness to the reference RDM. Only the highly expected face RDM differed from the alternative RDM testing for a u-shaped representation of presented faces (black horizontal line on top in Fig. 4b).

## Discussion

Our findings demonstrate that face expectations are represented in the high-level face-sensitive region in the right aTL. During the presentation of scene cues, representations of expected faces, measured in form of multivoxel fMRI patterns in the face-sensitive aTL, contained a graded amount of face information depending on the strength of the corresponding face prior. Conversely, during the presentation of unexpected face images, activity increased in a typical distributed network for processing surprise and prediction error^33,34^. Our work contributes to our understanding of how the strength of perceptual expectations is represented by supporting the assumption that the strength or certainty of anticipated stimuli is coded with the pre-activated stimulus representation. In line with previous research mostly done on lower-level visual stimuli such as gratings or stimulus categories, our findings indicate that anticipated stimuli are pre-activated in the corresponding sensory brain area^16–20^. We extended this previous work by investigating the anticipation of face images and could show that when several faces are expected with different probabilities, corresponding pre-activations of these faces are simultaneously represented in the high-level face-sensitive aTL.

From previous work on representations of presented stimuli, we know that corresponding evoked univariate as well as multivariate activity patterns depend on how much these stimuli were expected. Increased univariate responses to surprising faces have been repeatedly shown in the FFA^7–9,38^. For example, a previous univariate fMRI study demonstrated sensitivity to the conditional probabilities of face stimuli in the FFA on a categorical level (i.e., face category vs house category), reporting the summation of activity related to prediction (face expectation) and prediction error (face surprise) in blocks with different probabilities for seeing a face vs a house (25% and 50%, respectively)^8^. The results from previous multivariate analyses are more controversial^12^. Different methodological approaches found a dampening of stimulus representations by expectations, e.g., with reduced decoding of expected compared to unexpected stimuli in monkey inferotemporal cortex^39^ and scaled expectation suppression magnitude depending on image preference also in monkey inferotemporal cortex^40^ and in the human visual cortex^41^. In contrast, others found a sharpening of representations by expectations, e.g., increased encoding of expected grating orientations^42^ and of visual displays congruent with sensorimotor predictions^43^ despite reduced univariate responses, and increased decoding of expected faces from single-unit recordings in monkey inferotemporal cortex^44^.

In our study, we could also test the evoked univariate fMRI response to presented faces since they were either expected with low, mid, or high probability. The only univariate modulation effect in the FFA was for a u-shaped response, i.e., increased activity for the highly expected and unexpected faces in comparison to the intermediate condition (Fig. 3c). This u-shape response to presented faces modulated by probability could reflect that in FFA two processes take place simultaneously, i.e., enhanced processing of highly expected faces, as well as enhanced prediction error responses to unexpected faces^45^, similar to effects of probability on memory^35^. The corresponding measured fMRI signal may not combine in a linear way, leading to the observed univariate quadratic effect. Furthermore, unexpected faces evoked activity in a network typically associated with surprise, task control, action evaluation, and general attentional mechanisms including bilateral caudate, anterior insula, middle frontal gyrus, parietal lobe, precuneus, and superior frontal gyrus (Fig. 3a^33,46,47^). The linear increase in activity in these regions for unexpected faces could reflect response adjustment and task difficulty since participants potentially had to change their planned response upon the presentation of an unexpected face. Finally, expected faces activated the medial frontal cortex (Fig. 3b), a region involved in outcome monitoring^48^.

In our design, participants were asked to learn the underlying statistical regularities and perform a task to explicitly evaluate the probability of the presented stimuli, likely resulting in explicit/intentional learning. Therefore, our design intentionally differed from many previous studies investigating the role of sensory priors, which used visual statistical learning to form sensory priors, mostly relying on many repetitions and incidental learning^40–43,49^. These differences may also, at least partially, account for differences in our results compared to prior studies; i.e., the present results show little univariate differences between expected and unexpected stimuli in the sensory cortex, while numerous previous studies show extensive suppression throughout sensory areas to expected stimuli (e.g.,^41,49,50^). Another discrepancy between the present and previous studies is the long interval between cue and stimulus, a necessary adjustment for our fMRI study to assess pre-stimulus activity. In this extended pre-stimulus time window, participants may have engaged in mental imagery, already imagining the most likely upcoming stimulus. Prior work showed that imagery can result in similar representational patterns and relies on similar top-down connectivity as perception^51,52^. It is an open question whether and how cue-based mental imagery and explicit/intentional expectations refer to different processes or indeed rely on the same neural substrates for selective top-down activation of sensory cortex^53^. From philosophical perspectives, it has been suggested that mental imagery provides a way to generate explicit predictions and simulate sensory input given different situations^54,55^.

In addition, our study enabled us to apply a multivariate approach to test expectation-weighted representations of presented faces. We aimed to go beyond categorical face expectations and to test how individual face images are expected and potentially differentially weighted according to their probability. In contrast to previous studies investigating categorical face processing^7,8^, we did not find evidence for either a graded representation of presented or expected faces in the FFA, in line with other studies investigating face identity (independent of prior expectations^56^). To rule out that we were not able to detect face representations due to inter-individual variability in FFA localization, we also localized posterior and anterior FFA on individual statistical maps. One explanation for the absence of expectation-dependent face representations in FFA in our study is that previous studies identifying expectation effects for faces in FFA manipulated the probability of categorical face priors (i.e., face vs house^8^), whereas we induced expectations for three different face images in parallel.

We also tested for representations of presented faces that increase with expectedness, i.e., a potential sharpening or confirmation effect of a predicted face image. Only in the low-level OFA, we identified enhanced representations, i.e., higher correlations between response patterns evoked by the face image from the localizer with the corresponding presented face image in the test runs that was expected with high probability (Fig. 4b). Previous studies suggested that while both OFA and FFA both discriminate between faces, both may serve different computational roles and the face-related information encoded in OFA and FFA may be distinct:^57^ While OFA responds to differences in low-level image-based properties, FFA represents higher-level perceptual and social face information, which was not associated with our face images in the current study. Hence, the increased representation for highly expected presented faces may reflect sharpened low-level facial features in OFA.

The main goal of this study was to go beyond the indirect inference based on responses to presented stimuli to test whether expectation strength is represented in sensory processing areas before a stimulus is presented. Previous work provides evidence for prior representations in form of pre-activations of expected stimuli with different neuroimaging methods (fMRI:^16,17^, MEG:^18,19^, and intracranial cortical recordings^20^). However, it is still highly debated how the strength of expectations is represented in the human brain^22,58^. According to hierarchical predictive processing, expectation strength could be explicitly represented at all stages along the processing hierarchy, including sensory areas.

Our results provide evidence that during face expectations multivariate fMRI patterns in the high-level face-sensitive right aTL contained a graded amount of face information depending on the strength of the corresponding expectation. This finding provides further empirical evidence for computational architectures like predictive coding and supports a hierarchical organization of face-processing beyond pure feedforward schemes^2,8^. The face area on the ventral surface of the right aTL in or near the perirhinal cortex has been repeatedly implicated in facial processing^24,26,59^ and representing face identity at a high level within the face-processing hierarchy in the temporal lobe^27–29,56,60^. Face-sensitive regions within the temporal lobe form a tightly interconnected network in which the face-sensitive aTL interacts and modulates activity in the FFA^61,62^. Therefore, the face-sensitive aTL is ideally suited to send information about weighted face-priors to lower-level face-sensitive regions, such as the FFA, to improve context-dependent processing of incoming face information.

Our design and stimulus set was strictly controlled do detect changes in fMRI response patterns in response to strength-dependent face expectations, but we acknowledge that the presented work is not without limitations. Firstly, in our controlled stimulus set and design, we did not test viewpoint-independent representations of face identity, as we were using only one face image per identity (as in ref. ^63^). A design using different pictures of the same people would have facilitated a conclusion about representation of image-independent facial identity. We only used a set of three computer-generated faces allowing us to control that these faces were perceived as equally distant from each other (see pilot study in Supplementary Information, Supplementary Fig. 1). A more naturalistic approach of measuring face identity representations has been taken in studies using videos^57^ and it would be interesting to investigate the impact of expectation and context on these.

Secondly, the relatively small effect sizes that we have observed are common for multivariate fMRI analyses of individual stimuli. The maximum possible correlation values that could be observed in our fMRI data from the aTL [ceiling: 0.1056 0.2019] and the OFA [ceiling: 0.0806 and 0.1593] form upper bounds that are substantially smaller than 1 (see Fig. 4), indicating limitations of our experimental data (e.g., low spatial resolution, high measurement noise, and/or limited amounts of data). Nonetheless, none of these limitations should differentially affect our experimental conditions and hence measurement noise or other extraneous factors cannot explain the observed similarity effects seen in the multivariate analyses. For RSA, similar correlation values between fMRI-response based- and hypothesized similarity matrices have been observed previously^64–67^. Similarly, low classification accuracies are also common in decoding task events using Multivariate Classification of fMRI data^68,69^. Despite these small effect sizes, condition-specific differences in the observed correlations (i.e., the different amounts of similarity across the different levels of expectation strength) provide compelling support for expectation-dependent multivoxel representations. However, the cross-subject consistency measures suggest that with a more appropriate hypothesis RDM we could have improved the correlation values obtained with our theoretically motivated hypothesis RDM.

Finally, we restricted the regions of interest (ROIs) to the face-sensitive regions in the ventral stream from the occipital and fusiform face area (OFA and FFA) to the face-sensitive region in the aTL^23,24,26–29,56^. Notably, though we focused on face-sensitive regions in the right hemisphere, because activations in face regions are often stronger, more consistent, and larger in the right hemisphere^30,56,57,70^, analogous to observations from brain lesions that the right hemisphere is specialized for face processing^71,72^, similar face-related activations were found in the left hemisphere and we do not rule out that these also play a role in processing and generating face expectations. Furthermore, faces are processed in several other regions outside the ventral stream, for example, in the frontal lobe^73,74^ or the superior temporal sulcus (STS), which responds stronger to dynamic than static face stimuli^75–77^. An exploratory analysis of representations of expected and presented faces, measured in form of multivoxel fMRI patterns in the STS, did not reveal any influence of face expectations in the current study (see Supplementary Note 1). Potentially, these regions process context- and experience-derived expectations with respect to other aspects of facial information, such as priors for gaze or facial expressions in the STS^78^. The present study offers a basis for targeting these questions in the future.

In conclusion, our results indicate that during face expectations the face-sensitive aTL contained a graded amount of face information depending on the strength of the corresponding prior. This finding supports theories of predictive coding which suggest that the expectation strength is co-localized with the representation of the prior itself^2,8^. In addition, there seem to be additional specialized brain regions such as the caudate and the insula that represent surprise during the presentation of unexpected stimuli. Our study contributes to the notion that the brain is not a passive stimulus-response system, but rather an active, hierarchical system, in which anticipatory activity is weighted according to its prior probability^4^.

## Methods

### Participants

Thirty-five volunteers (16 female/19 male, age range 18–32 years) participated in the study. Written informed consent was obtained from all participants. All experimental procedures were approved by the Ethics Committee of the Chamber of Physicians in Hamburg. Five participants were excluded because of their behavioral performance indicating that they did not learn the scene-face contingencies (see Supplementary Fig. 2). The remaining 30 participants (15 female/15 male, age range 18–31 years) were included in the reported analysis.

### Stimuli

In the main experiment, we used images of three scenes and three faces. The images of the three scenes were selected to be different in color and site (i.e., blue beachfront, yellow field, and green grass, Fig. 1b) so that participants could discriminate them quickly and remember them easily. The scene images were normalized by their mean amplitude^79^. The three male face images were generated with FaceGen software (FaceGen Modeller 2.0, Singular Inversion) and selected based on a preceding behavioral face selection experiment (with different participants, *n* = 10, see next paragraph for details).

In the first step, eight male faces were generated with FaceGen. These faces were designed to present eight identities that were easy to differentiate based on features that have been shown to make faces distinct, such as lip thickness, eye color, and eye shape^80^. The goal of the face selection experiment was to select three face images, which were maximally and equally distinct from each other, from the set of these eight images. In a behavioral pre-study (with different participants, *n* = 10), at a given single trial (total 420 trials) a quadruple consisting of two pairs of faces (Fa–Fb and Fc–Fd) was presented and participants were required to select the pair that consisted of the more similar faces. The perceptual experiment was self-paced; however, the stimuli were turned off after 7 s, and participants were required to answer. The given responses were transformed into a representational dissimilarity matrix containing the perceptual distance between the eight face images. To do this, we filled the cells of the RDM with the number of dissimilar responses for each pair of faces and normalized them by the number of all responses. We used maximum likelihood difference scaling on the RDM averaged over all participants to select three face images that were perceived as equally distant from each other (Supplementary Fig. 1).

### Overview of the experimental protocol

The experiment started with a functional localizer in the scanner (30 min), followed by a training session outside the scanner (40 min), and finally the main scanning session which consisted of three test runs (60 min) (Fig. 1a). Across participants, we included three different combinations in which scene and face images were paired, so that each scene predicted each face identity with low, mid, and high probability in one of the versions. Participants were randomly assigned to one of the three versions.

In the functional localizer, participants saw images of three faces and three scenes before they had learned any associations between these images. The task was to detect red dots occasionally presented on the face images. The localizer contained 9 catch trials with a task in addition to 72 trials (eight repetitions of three scenes paired with three faces) and lasted 16.8 min. The task was included to make sure that participants paid attention to the face images. Across participants, detection performance was high (mean correct 0.96, with a standard deviation of 0.19). Responses of one participant were not recorded during the localizer. The functional localizer run was used for was used for ROI definition with a univariate contrast and for the RSA.

Participants were trained to relate images of scenes and faces outside the scanner. Each scene predicted three faces: one with low, one with intermediate, and one with high probability (10, 30, or 60%). First, we provided written instruction with explicit information about the corresponding probabilities between scene and face images. Second, participants experienced these probabilities in a training block in which scene and face images were paired according to their associated probabilities (20 repetitions for each of the three scenes, resulting in 60 trials of 5 min duration). Here, to facilitate learning, the scenes were presented in blocks. Third, participants viewed scenes followed by the face images and had to indicate in a Three-Alternative-Forced–Choice Task (3AFCT) for each face whether that face was expected with low, intermediate, or high probability, given the preceding scene (30 repetitions for each of the three scenes, resulting in 90 trials of 10.50 min). Participants received feedback about whether their response was correct, incorrect, or too slow. In the final training part, participants performed the same test again, while they were familiarized with the timing in the scanner, which contained jittered and longer inter-stimulus intervals (10 repetitions for each of the three scenes, resulting in 30 trials of 7 min). In this final part, participants only received feedback about whether their response was too slow.

The test phase was recorded with fMRI to measure face expectations and their associated strength during scene presentation. Participants performed the 3AFCT and indicated via button press whether a presented face was expected with low, intermediate, or high probability (Fig. 1a). There were three functional runs.

The first and the third run were identical. Participants only received feedback about too slow responses. Importantly, these two runs were extinction runs, in which faces were presented with equal contingencies after each scene. This was done to avoid that functional measurements of expected faces during scene presentation could be confounded by the following presented faces. In the second, middle functional run, participants received another training block with feedback about correct, incorrect, and too slow responses. In this second run, the contingencies followed the trained/expected contingencies between scene and face images.

The trial timing was identical in all functional runs. The scene image was presented for 500 ms, followed by a jittered fixation cross of 4–8 s (6 s on average), followed by a face presented for 1 s. Finally, either a fixation cross (localizer, run 1 and 3) or feedback (correct, incorrect, or too slow in run 2; too slow in runs 1 and 3) was presented for 500 ms. The inter-trial interval was jittered for 4–8 s (6 s on average). In runs 1 and 3, the combination of three faces and three scenes was repeated 16 times, resulting in a run with 72 trials of 16.8 min. In run 2, there were 20 repetitions per scene, resulting in a run with 60 trials of 14 min.

### MRI data acquisition

All imaging data were acquired on a Prisma 3 T scanner (Erlangen, Germany) using a 64-channel head coil. Functional data were obtained using a multiband echo-planar imaging sequence (repetition time (TR) = 0.967 s, echo time (TE) = 30 ms, flip angle = 50°, field of view (FoV) = 224 mm, multi-band mode, number of bands: 3, interleaved phase encoding in descending order). Each volume of the experimental data contained 45 slices (voxel size 2 × 2 × 2 mm + 0.5 mm gap).

An additional structural image (magnetization prepared rapid acquisition gradient echo) was acquired for functional preprocessing and anatomical overlay (TR = 0.230 s, TE = 0.298 s, flip angle = 9°, FoV = 256 mm, 240 slices, voxel size 1 × 1 × 1 mm).

A fieldmap was acquired (TR = 0.555 s, TE 1 = 0.612 s, TE2 = 0.858 s, flip angle = 40°, FoV = 224 mm, 45 slices (voxel size 2 × 2 × 2 mm + 0.5 mm gap). The detailed scanning protocols are available here https://osf.io/uygvm/.

Participants viewed the back-projected stimuli via a 45° mirror placed atop the head coil, images were presented on the screen InroomViewingDevice (www.nordicneurolab.com/product/inroomviewing-device). The stimuli were presented with the Psychtoolbox (http://psychtoolbox.org) in MATLAB.

### Behavioral analysis

To test whether participants learned the instructed contingencies and associated the three scenes and faces with the corresponding low, intermediate, and high probabilities, we computed mean correct responses averaged across test runs 1 and 3 for the three levels of probability. As inspection of individual correct performance indicated that participants differed in their response profiles, we visualized these profiles by grouping participants based on whether a linear or quadratic polynomial curve provided a smaller sum of squares due to error as a goodness-of-fit measure. This approach was descriptive. In addition, we tested whether reaction times differed by a Repeated Measures Single-Factor Analysis of Variance Test.

### fMRI data analysis preprocessing

Structural and functional data were analyzed using SPM12 (Welcome Department of Cognitive Neurology, London, UK) and custom scripts in MATLAB. First, we applied field mapping distortion correction to the functional volumes to correct for geometric distortions in EPI caused by magnetic field inhomogeneity (with the FieldMap toolbox). The functional images of all runs were realigned and the individual structural T1 image was co-registered to the mean functional image generated during realignment. The functional images were spatially normalized to MNI space. For the univariate analysis, the functional images were additionally smoothed with a 6 mm full-width at half maximum isotropic Gaussian kernel.

### Univariate fMRI analysis

Data of all three functional runs (i.e., the functional localizer and the two test runs without feedback) were analyzed using the general linear model (GLM) with a 128 s high pass filter. We applied SPM’s alternative pre-whitening method to account for autocorrelation, FAST, which has been suggested to perform better than SPM’s default^81^. Raw motion parameters (three translations and three rotations), their derivatives, squared derivatives, as well as the average time-course of the left and right ventricles were included as nuisance covariates.

A functional localizer run was recorded before the training to define face-sensitive ROIs (FFA and aTL)^28,56^. We included the onset of 7 event types in the GLM, each convolved with the canonical SPM haemodynamic response: 7 conditions come from specifying the onset of three scenes, three faces, and the onset of the task in catch trials. On the second level, we computed the *t*-contrast ‘face images > scene images’ to localize face-sensitive regions (see Supplementary Table 1). In addition, we computed 6 separate contrasts of all three individual face and scene images against the baseline to obtain beta-images for the following RSA analysis (see below).

The GLM for the first and last run of the test phase in the scanner contained the onsets of 13 event types in the GLM, each convolved with the canonical SPM haemodynamic response: 13 conditions come from specifying the three repressors for each of the three scenes (corresponding to three sets of simultaneous face expectations: low probability for Face 1/2/3 + intermediate probability for Face 1/2/3 + high probability for Face 1/2/3, respectively); three repressors for each of the three presented faces weighted by whether they were expected with low, intermediate or high probability (i.e., 9 face onsets), and one onset for ‘too slow’ feedback. In sum, for the subsequent RSA analysis, we used one regressor per condition, i.e., 3 scene regressors for expectation, and 3 × 3 face regressors for presentation.

In addition, to investigate the effect of face expectation strength on the activation during face presentation, we set up a GLM with a parametric modulator on the presented faces. Specifically, we tested for (1) an increased signal in response to presented faces that were less expected by means of a negative linear parametric modulator, (2) an increased signal in response to presented faces that were more expected with a positive linear parametric modulator, and (3) a u-shaped univariate response reflecting increased activity for highly expected and unexpected faces with with a quadratic parametric modulator. On the second level, we tested for the linear modulator whether it was larger or smaller than 0, respectively, and for the quadratic modulator whether it was larger than 0.

### ROI definition

We defined the face-sensitive ROIs based on the functional localizer run before participants learned the associations between face and scene images (see Supplementary Table 1). We aimed to localize the higher-level face-sensitive region in the aTL, a hierarchically-connected face-sensitive region that processes face identity, as well as the occipital and posterior and anterior fusiform face area (OFA, pFFA, aFFA)^23,24,26–29,56^ (see Supplementary Fig. 3). For the face-sensitive region in the right aTL, we obtained the cluster size k = 776, peak at [39, −18, −32] from the contrast ‘face images > scene images’ at *p* < 0.001, uncorrected and masked with the right temporal fusiform cortex anterior division from the Oxford Harvard atlas. For localization of the OFA and the posterior and anterior FFA, we combined the contrast ‘face images > scene images’ with the three respective clusters from the atlas^82^. We used different levels of uncorrected threshold to obtain non-overlapping clusters. We obtained for OFA a peak at [32, −74, −16], k = 934, at *p* < 0.0001, a sphere of 6 mm around the peak for the posterior FFA at [39, −52, −10], k = 264, and for the anterior FFA at [42, −34, −19], k = 254. We chose this size to obtain two non-overlapping ROIs for the anterior and posterior FFA (see Supplementary Fig. 3).

To not overlook any effects because of differences in individual localization of the posterior and anterior FFA across participants^70^, we also extracted individual pFFA and aFFA ROIs from the individual statistical localizer maps (face images > scene images). Specifically, we used the aFFA and pFFA ROI from the second level and searched for the peak maximal 6 mm away from this ROI in each participant’s individual statistical *t* map. From that individual peak we again extracted an individual ROI with a sphere of 6 mm.

As in our previous work, we explicitly focused on face-sensitive regions in the right hemisphere^28,83^, in line with the consideration that face-selective regions are more consistent and larger in the right hemisphere^57,70^. Furthermore, high-level face-identity processing in the aTL has predominantly been shown in the right hemisphere, when both hemispheres were tested^30,56^.

### Representational similarity analysis

We used RSA to test whether multivariate pattern similarity between presented and expected faces depends on the prior strength^37^.

For each of the three scene and the three face item-specific regressors, we estimated single-subject beta-statistic images for the contrast of image onset compared to the unmodelled resting period, for each of the three scanning runs (localizer and the two extinction test runs). We used the resulting single item-specific T-images for RSA^36^ using the RSA toolbox^37^.

RSA involves testing whether the observed similarity of brain responses in specific conditions (a neural representational dissimilarity matrix, i.e., the reference RDM) corresponds to a hypothetical pattern of similarity between these conditions (hypothesis RDM). For that, we designed hypothesis representational dissimilarity matrices (RDMs) that tested for similarity of (1) expected faces and (2) presented faces, depending on the strength of the expectation (visualized at the bottom of Fig. 4a and b, respectively). As in previous studies^67,84,85^, we used subsections to test our hypotheses about representational similarity across localizer and test runs. Specifically, to evaluate how representational dissimilarity depends on prior strength, we weighted the hypothesized similarity (1) with the three graded levels of expectation strength (i.e., 1, 0.5, 0), (2) the highly expected face (i.e., 1, 1, 0), and (3) the face with the low and high probability (i.e., 0, 1, 0) for the expected faces (Fig. 4a) and the presented faces (Fig. 4b), respectively. We measured multivoxel RDMs by computing the dissimilarity (1–Pearson correlation across voxels) of T-statistics for each either presented or expected weighted face image. The similarity between the observed RDM in each ROI and each of the hypothetical RDMs was computed using Kendall’s tau A correlation coefficient because of the tied rank for the ‘only high expected face’ RDM (as implemented in ref. ^37^). For comparing dissimilarity matrices, we prefer correlation distance, because it is invariant to differences in the mean and variability of the dissimilarities and since we do not assume a linear match between dissimilarity matrices.

The relatedness of each hypothesis RDM to the reference RDM was measured as the average across subjects of the correlations between the hypothesis RDM and the single-subject reference-RDM estimates (as implemented in ref. ^37^). The relatedness of each hypothesis RDM to the reference RDM (bar height in Fig. 4) was tested using a one-sided signed-rank test across the single-subject RDM correlations.

To test whether two hypothesis RDMs differ in their relatedness to the reference RDM, the difference between the RDM correlations in each subject was computed and a two-sided signed-rank test across subjects was performed. This procedure was repeated for each pair of RDMs and multiple testing was accounted for by controlling the false-discovery rate. The significant comparisons are indicated by horizontal lines above the bars (Fig. 4).

To provide an estimate of the maximum possible correlation value between the observed RDM and the hypothesized RDMs, we used the procedure described in^37^ for computing the upper bound of the noise ceiling of the observed RDMs for the fMRI data. Specifically, the rank-transformed single-subject RDMs were averaged and were used in an iterative procedure to find the RDM with the maximum average correlation to the single-subject RDMs (using published code from^37^).

In addition, to provide an estimate of the expected correlation value between the observed RDM and the hypothesized RDMs, given the degree of inter-subject variation in the fMRI data, we computed the cross-subject consistency of the observed RDMs (using the procedure described for computing the lower bound of the noise ceiling in^37^ and the corresponding published code). Specifically, a leave-one-subject-out procedure was used in which each subject’s empirically observed RDM was correlated using Kendall’s Τau A coefficient with the mean observed RDM of the remaining 29 subjects. The empirical reference RDMs were computed within the ROIs.

### Statistics and reproducibility

We recruited 35 participants and ended up including 30 participants as we had to exclude five participants because of their behavioral performance indicating that they did not learn the scene-face contingencies (see Supplementary Fig. 2).

The univariate fMRI analyses in the whole brain were *p* < 0.05 cluster FWE-corrected with a *p* < 0.001 inducing threshold. In the region of interest analysis, we used FWE correction at the peak level.

In the multivariate fMRI analysis, the correlation between the reference RDM and the hypothesis RDMs was used to measure the performance of each hypothesis RDM and significance was evaluated with FDR control *p* < 0.05. To test whether two hypothesis RDMs differed in their relatedness to the reference RDM, the difference between the RDM correlations in each subject was computed and a two-sided signed-rank test across subjects was performed. This procedure was repeated for each pair of RDMs and multiple testing was accounted for by controlling the false-discovery rate.

### Reporting summary

Further information on research design is available in the Nature Portfolio Reporting Summary linked to this article.

## Supplementary information

Supplementary Information-New

Reporting Summary

## Data Availability

The experimental data that support the findings of this study are available from https://osf.io/uygvm/10.17605/OSF.IO/UYGVM.

## Code availability

The code that supports the findings of this study is available from https://osf.io/uygvm/10.17605/OSF.IO/UYGVM.
