# Supplementary material for: Multivariate functional neuroimaging analyses reveal that strength-dependent face expectations are represented in higher-level face-identity areas
Source: Commun Biol. 2023 Feb 1;6:135. doi: 10.1038/s42003-023-04508-8 (PMC9892564; doi:10.1038/s42003-023-04508-8)
Supplement: Supplementary file 1 — Supplementary Information-New [file 42003_2023_4508_MOESM1_ESM.pdf]

Supplementary Information: Multivariate functional neuroimaging analyses reveal that strength-dependent face expectations are represented in higher-level face-identity areas

Authors: Helen Blank[1*], Arjen Alink[1] & Christian Büchel[1]

[1]Department of Systems Neuroscience, University Medical Center Hamburg-Eppendorf, Hamburg 20246, Germany.

Supplementary Information

Supplementary Figure 1. MDS Selection of three face images from behavioural pre-experiment.

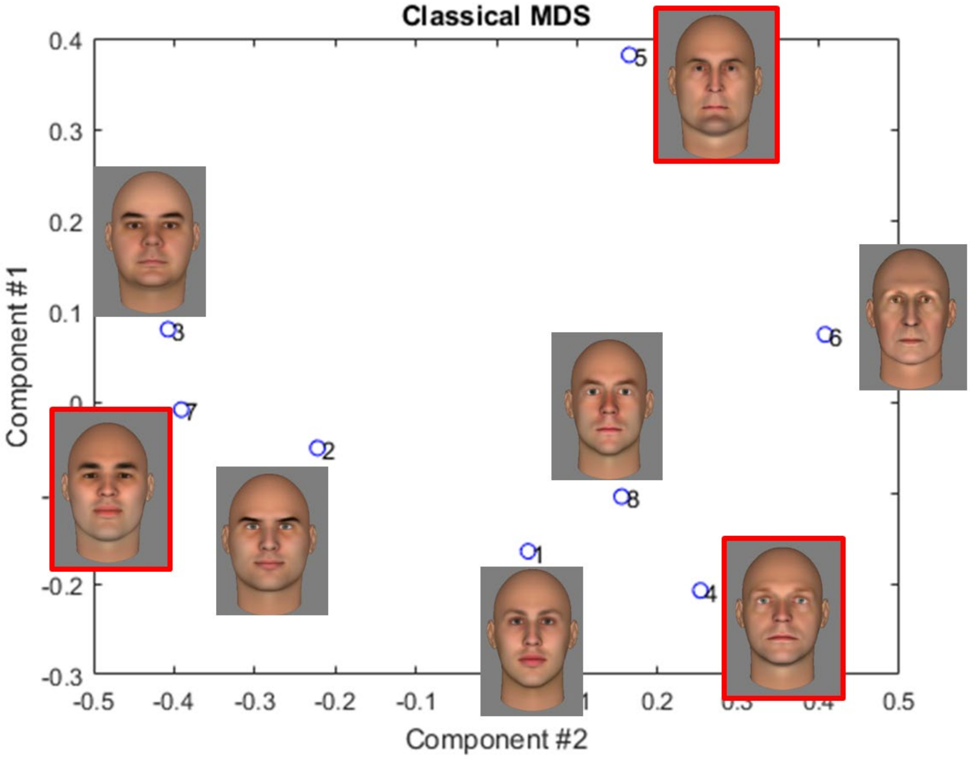

We conducted a behavioural pre-study (with different participants, n = 10) to select three face images from a set of eight images, which were maximally and equally distinct from each other. To obtain an RDM with the perceptual distance between the eight face images, the number of dissimilar responses for each pair of faces was normalized by the number of all responses. We used maximum likelihood difference scaling (MLDS) on the RDM averaged over all participants to select three face images that were perceived as equally distant from each other.

Supplementary Figure 2. Individual participants' behavioural responses

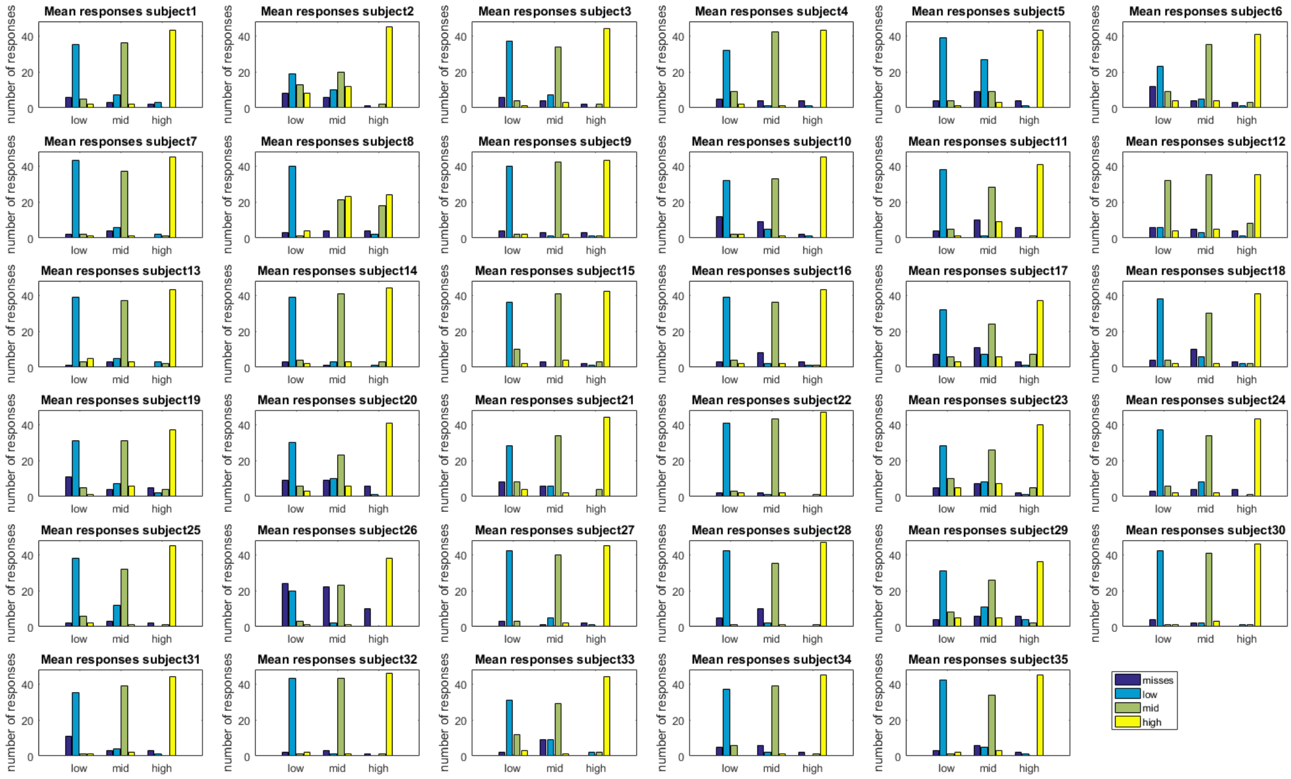

Behavioural responses show that most of the participants correctly associated scene cues and face images based on the three different probabilities (low in blue, mid in green, and high in yellow). Participants, 5, 8, 12, and 26 were excluded because of poor performance (i.e., less than 60 % correct across all three conditions or less than 20 % in a single condition).

Supplementary Figure 3. Localizer for face-sensitive regions.

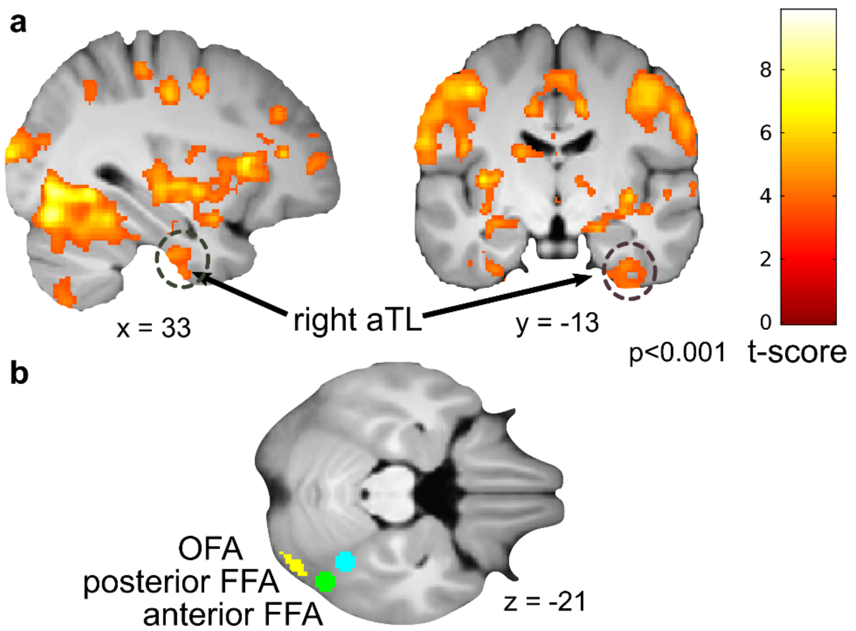

The univariate contrast “faces > senses” from the first localizer run was used to localize the face-sensitive regions of interest in the right hemisphere **a)** in the right anterior temporal lobe (in A depicted at p<0.001, uncorrected), **b)** in the posterior and anterior fusiform face area (FFA), and the occipital face area (OFA, in B, all three regions were masked with the respective clusters from a probabilistic atlas containing OFA and the posterior and anterior FFA [1]).

Supplementary Table 1. Localizer Faces > scenes, p < 0.001, uncorrected + cluster-corrected p(FWE) < 0.05

cluster p(FWE-corr)	cluster equivk	peak p(FWE-corr)	peak T	peak equivZ	x,y,z {mm}	label
<0.001	43635	0.000	9.84	6.47	28 -73 -8	occipital fusiform gyrus
		0.000	9.47	6.35	12 -98 4	
		0.000	9.31	6.29	12 -80 -12	
<0.001	13200	0.005	7.40	5.50	-22 -1 -20	amygdala
		0.009	7.17	5.39	-34 -14 6	
		0.010	7.14	5.38	-40 14 -8	
<0.001	15162	0.007	7.30	5.46	33 18 10	frontal operculum
		0.014	6.98	5.30	27 -1 -19	
		0.029	6.64	5.14	32 -4 -6	
<0.001	6709	0.010	7.13	5.38	6 11 34	middle cingulate gyrus
		0.013	7.01	5.32	8 2 36	
		0.032	6.60	5.12	-10 18 40	
0.003	405	0.070	6.24	4.93	20 47 -14	medial orbital gyrus
		0.627	5.08	4.26	24 32 -14	
<0.001	858	0.322	5.50	4.51	-30 46 2	medial/posterior orbital gyrus
		0.690	5.00	4.21	-18 50 8	
		0.955	4.55	3.92	-30 56 4	
<0.001	640	0.400	5.37	4.44	27 -62 -54	middle frontal gyrus
		0.988	4.38	3.81	33 -67 -56	
		0.989	4.37	3.80	20 -68 -56	
0.003	408	0.496	5.24	4.36	-32 -10 -34	fusiform gyrus
		0.658	5.04	4.24	-40 -19 -26	
		0.976	4.46	3.86	-32 -8 -43	
<0.001	921	0.500	5.24	4.36	-10 -76 -43	cerebellum
		0.909	4.67	4.00	-8 -78 -36	
		0.936	4.61	3.96	-28 -74 -48	

Supplementary Table 2. Univariate analysis of presented faces with linear parametric modulator in run 1+3, p < 0.001, uncorrected + cluster-corrected p(FWE) < 0.05

cluster p(FWE-corr)	cluster equivk	peak p(FWE-corr)	peak T	peak equivZ	x,y,z {mm}	label
<0.001	1742	0.000	9.11	6.21	-32 22 -2	anterior insula
		0.974	4.48	3.88	-48 17 -10	
		0.975	4.48	3.87	-45 16 5	
<0.001	2713	0.000	8.65	6.04	33 23 -4	anterior insula
		0.002	7.78	5.68	42 20 -6	
		0.004	7.51	5.55	34 22 -13	
<0.001	4258	0.001	8.33	5.91	36 -74 42	middle occipital gyrus
		0.006	7.40	5.50	38 -43 41	
		0.008	7.22	5.42	33 -64 47	
<0.001	2706	0.001	8.00	5.77	51 23 28	middle frontal gyrus
		0.003	7.64	5.61	46 32 26	
		0.242	5.66	4.61	48 16 30	
0.001	497	0.004	7.55	5.57	-10 -78 -30	cerebellum
<0.001	3149	0.012	7.07	5.35	4 -68 48	precuneus
		0.354	5.46	4.49	0 -62 40	
		0.421	5.35	4.43	6 -66 35	
<0.001	2621	0.012	7.06	5.34	-40 20 26	middle frotnal gyrus
		0.217	5.71	4.64	-50 26 30	
		0.346	5.47	4.50	-44 5 38	
<0.001	2594	0.027	6.68	5.16	-34 -54 42	angular gyrus
		0.038	6.53	5.08	-34 -44 40	
		0.678	5.03	4.23	-44 -42 42	
<0.001	2597	0.117	6.01	4.81	8 18 46	supplementary motor area
		0.173	5.83	4.70	2 30 44	
		0.402	5.38	4.45	-4 17 48	
<0.001	679	0.119	6.01	4.80	-34 -73 -54	cerebellum
<0.001	635	0.139	5.93	4.76	9 5 0	caudate
		0.957	4.55	3.92	12 14 4	
		1.000	4.12	3.62	12 -4 -2	
0.027	292	0.212	5.73	4.65	8 -25 -4	ventral DC
		0.339	5.48	4.50	-3 -30 -2	
		0.945	4.59	3.95	12 -12 6	
0.009	367	0.352	5.46	4.49	33 -73 -55	cerebellum
0.005	407	0.533	5.21	4.34	9 -85 -38	
		0.902	4.70	4.02	9 -79 -32	
		1.000	4.00	3.54	15 -78 -25	
<0.001	758	0.621	5.10	4.27	-50 41 0	inferior frontal gyrus

		0.972		4.49	3.88	-39 48 5	
			1.000	3.91	3.47	-46 52 -6	
0.003	443	0.717		4.98	4.20	60 -38 -13	middle temporal gyrus
			1.000	4.09	3.61	69 -48 -8	
0.014	335	0.747		4.94	4.17	22 62 -14	anterior orbital gyrus
			1.000	3.79	3.39	12 65 -10	
0.066	237	0.783		4.89	4.14	-8 8 -1	caudate
		0.822		4.83	4.11	-8 2 5	
0.002	471	0.818		4.84	4.11	34 2 44	middle frontal gyrus
		0.956		4.56	3.93	33 6 54	
		0.995		4.31	3.76	28 12 50	
0.041	266	0.945		4.59	3.95	2 -60 -42	cerebellar ventral lobules
		0.994		4.32	3.77	-4 -58 -50	
		0.997		4.26	3.72	-8 -56 -42	

Supplementary Note 1. Results for the Superior Temporal Sulcus (STS)

In addition to the face-sensitive regions in the ventral stream, we explored expectation-dependent face representations in the superior temporal sulcus (STS). In analogy to the localization of the other ROIs, we obtained the STS cluster of size k = 166 with a peak at [45, -26, -2] from the contrast “face images > scene images” at $p < 0.001$, uncorrected and masked with the right superior temporal cortex from the Oxford Harvard atlas.

In the STS, multivariate patterns of presented faces in the localizer did not correlate with multivariate patterns of expected faces during scene presentation in the test runs (u-shape relationship: $r = -0.0056$, $Z(29) = -0.3312$, $p = 0.6298$; only highly expected face: $r = -0.0130$, $Z(29) = -0.9620$, $p = 0.8320$; graded face presentations: $r = -0.0148$, $Z(29) = -0.2995$, $p = 0.6177$). The corresponding hypothesis RDMs did not differ in their relatedness to the reference RDM and the corresponding noise ceiling in the STS was [0.0370 0.1296].

Multivariate patterns of presented faces in the localizer did also not correlate with multivariate patterns of presented faces in the test runs (u-shape relationship: $r = 0.0630$, $Z(29) = 1.6283$, p =

0.0517; only highly expected face: r = 0.0093, Z(29) = 0.6892, p = 0.2454; graded face presentations: r = -0.0315, Z(29) = 0.8384, p = 0.7991). The corresponding hypothesis RDMs did not differ in their relatedness to the reference RDM and the corresponding noise ceiling in the STS was [0.0787 0.1500].

Future experimental designs manipulating priors about facial movement, gaze, or emotion may be more powerful to investigate expectation-dependent face representations with respect to these aspects of facial information in the STS [2].

Supplementary References

1. Zhen, Z. *et al.* Quantifying interindividual variability and asymmetry of face-selective regions: A probabilistic functional atlas. *NeuroImage* **113**, 13–25 (2015).
2. Furl, N., van Rijsbergen, N. J., Treves, A., Friston, K. J. & Dolan, R. J. Experience-dependent coding of facial expression in superior temporal sulcus. *Proc. Natl. Acad. Sci. U. S. A.* **104**, 13485–9 (2007).
