# Supplementary material for: Multivariate functional neuroimaging analyses reveal that strength-dependent face expectations are represented in higher-level face-identity areas
Source: Commun Biol. 2023 Feb 1;6:135. doi: 10.1038/s42003-023-04508-8 (PMC9892564; doi:10.1038/s42003-023-04508-8)
Supplement: Supplementary file 2 — Reporting Summary [file 42003_2023_4508_MOESM2_ESM.pdf]

nature portfolio

Corresponding author(s): Helen Blank

Last updated by author(s): Jan 19, 2023

Reporting Summary

Nature Portfolio wishes to improve the reproducibility of the work that we publish. This form provides structure for consistency and transparency in reporting. For further information on Nature Portfolio policies, see our Editorial Policies and the Editorial Policy Checklist.

Statistics

For all statistical analyses, confirm that the following items are present in the figure legend, table legend, main text, or Methods section.

n/a	Confirmed	
☐	☒	The exact sample size (*n*) for each experimental group/condition, given as a discrete number and unit of measurement
☐	☒	A statement on whether measurements were taken from distinct samples or whether the same sample was measured repeatedly
☐	☒	The statistical test(s) used AND whether they are one- or two-sided<br>*Only common tests should be described solely by name; describe more complex techniques in the Methods section.*
☒	☐	A description of all covariates tested
☐	☒	A description of any assumptions or corrections, such as tests of normality and adjustment for multiple comparisons
☐	☒	A full description of the statistical parameters including central tendency (e.g. means) or other basic estimates (e.g. regression coefficient) AND variation (e.g. standard deviation) or associated estimates of uncertainty (e.g. confidence intervals)
☐	☒	For null hypothesis testing, the test statistic (e.g. *F*, *t*, *r*) with confidence intervals, effect sizes, degrees of freedom and *P* value noted<br>*Give P values as exact values whenever suitable.*
☒	☐	For Bayesian analysis, information on the choice of priors and Markov chain Monte Carlo settings
☒	☐	For hierarchical and complex designs, identification of the appropriate level for tests and full reporting of outcomes
☐	☒	Estimates of effect sizes (e.g. Cohen's *d*, Pearson's *r*), indicating how they were calculated

Our web collection on statistics for biologists contains articles on many of the points above.

Software and code

Policy information about availability of computer code

Data collection	Psychtoolbox was used for data collection.
Data analysis	Data were analysed using Matlab 2019. Imaging data were analysed using SPM12.

For manuscripts utilizing custom algorithms or software that are central to the research but not yet described in published literature, software must be made available to editors and reviewers. We strongly encourage code deposition in a community repository (e.g. GitHub). See the Nature Portfolio guidelines for submitting code & software for further information.

Data

Policy information about availability of data

All manuscripts must include a data availability statement. This statement should provide the following information, where applicable:

- Accession codes, unique identifiers, or web links for publicly available datasets
- A description of any restrictions on data availability
- For clinical datasets or third party data, please ensure that the statement adheres to our policy

The experimental data that support the findings of this study are available from https://doi.org/10.17605/OSF.IO/UYGVM.

Human research participants

Policy information about studies involving human research participants and Sex and Gender in Research.

Reporting on sex and gender	We did not perform sex/gender basead analyses given that we are interested in investigating neural mechanisms that are independent of participants' sex/gender.
Population characteristics	30 participants (15 female/15 male, age range 18 - 31 years) were included in the reported analysis.
Recruitment	Participants were recruited via advertisements for participation in an fMRI experiment.
Ethics oversight	All experimental procedures were approved by the Ethics Committee of the Chamber of Physicians in Hamburg.

Note that full information on the approval of the study protocol must also be provided in the manuscript.

Field-specific reporting

Please select the one below that is the best fit for your research. If you are not sure, read the appropriate sections before making your selection.

☐ Life sciences ☒ Behavioural & social sciences ☐ Ecological, evolutionary & environmental sciences

For a reference copy of the document with all sections, see nature.com/documents/nr-reporting-summary-flat.pdf

Behavioural & social sciences study design

All studies must disclose on these points even when the disclosure is negative.

Study description	Quantitative methods
Research sample	Thirty-five healthy volunteers (16 female/19 male, age range 18 - 32 years) participated in the study.
Sampling strategy	Participants were recruited via advertisements for participation in an fMRI experiment.
Data collection	The experiment started with a functional localizer in the scanner (30 min), followed by a training session outside the scanner (40 min), and finally the main scanning session which consisted of three test runs.<br>All imaging data were acquired on a Prisma 3T scanner (Erlangen, Germany) using a 64-channel head coil.
Timing	First fMRI: 07.08.2017<br>Last fMRI: 08.01.2018
Data exclusions	Five participants were excluded because of their behavioural performance indicating that they did not learn the scene-face contingencies (see Supplementary Figure 2). The remaining participants (15 female/15 male, age range 18 - 31 years) were included in the reported analysis.
Non-participation	No participant declined participation.
Randomization	Across participants, we included three different combinations in which scene and face images were paired, so that each scene predicted each face identity with low, mid, and high probability in one of the versions. Participants were randomly assigned to one of the three versions.

Reporting for specific materials, systems and methods

We require information from authors about some types of materials, experimental systems and methods used in many studies. Here, indicate whether each material, system or method listed is relevant to your study. If you are not sure if a list item applies to your research, read the appropriate section before selecting a response.

Materials & experimental systems

n/a	Involved in the study
☒	☐ Antibodies
☒	☐ Eukaryotic cell lines
☒	☐ Palaeontology and archaeology
☒	☐ Animals and other organisms
☒	☐ Clinical data
☒	☐ Dual use research of concern

Methods

n/a	Involved in the study
☒	☐ ChIP-seq
☒	☐ Flow cytometry
☐	☒ MRI-based neuroimaging

Magnetic resonance imaging

Experimental design

Design type

Event-related design

Design specifications

The test phase was recorded with fMRI to measure face expectations and their associated precision during scene presentation. Participants performed the 3AFCT and indicated via button press whether a presented face was expected with low, intermediate, or high probability. There were three functional runs.
The first and the third run were identical. Participants only received feedback about too slow responses. Importantly, these two runs were extinction runs, in which faces were presented with equal contingencies after each scene. This was done to avoid that functional measurements of expected faces during scene presentation could be confounded by the following actually presented faces. In the second, middle functional run participants received another training block with feedback about correct, incorrect, and too slow responses. In this second run, the contingencies followed the trained/expected contingencies between scene and face images.
The trial timing was identical in all three functional run. The scene image was presented for 500 ms, followed by a jittered fixation cross of 4 to 8 seconds (6 seconds on average), followed by a face presented for 1 second. Finally either a fixation cross (run 1 and 3) or feedback (correct, incorrect, or too slow in run 2, or too slow response in run 1 and 3) was presented for 500 ms. The inter-trial interval was jittered for 4 to 8 seconds (6 seconds on average). In run 1 and 3, the combination of three faces and three scenes was repeated 16 times, resulting in a run with 72 trials of 16.8 minutes. In run 3, there were 20 repetitions per scene, resulting in a run with 60 trials of 14 minutes.

Behavioral performance measures

We recorded button press and response time. Behaviour was analyzed with Matlab 2019b.

Acquisition

Imaging type(s)

functional and structural MRI

Field strength

3 Tesla

Sequence & imaging parameters

All imaging data were acquired on a Prisma 3T scanner (Erlangen, Germany) using a 64-channel head coil. Functional data were obtained using a multiband echo-planar imaging sequence (repetition time (TR)=0.967 s, echo time (TE)=30 ms, flip angle=50°, field of view (FoV)=224 mm, multi-band mode, number of bands: 3).
An additional structural image (magnetization prepared rapid acquisition gradient echo (MPRAGE)) was acquired for functional preprocessing and anatomical overlay (240 slices, voxel size 1 × 1 × 1 mm). Participants viewed the back-projected stimuli via a 45° mirror placed atop the head coil.

Area of acquisition

Each volume of the experimental data contained 45 slices (voxel size 2 × 2 × 2 mm + 0.5 mm gap).

Diffusion MRI ☐ Used ☒ Not used

Preprocessing

Preprocessing software

Structural and functional data were analysed using SPM12 (Welcome Department of Cognitive Neurology, London, UK) and custom scripts in MATLAB. First, we applied field mapping distortion correction to the functional volumes to correct for geometric distortions in EPI caused by magnetic field inhomogeneity (with the FieldMap toolbox). The functional images of all runs were realigned and the individual structural T1 image were co-registered to the mean functional image generated during realignment. For the univariate analysis the functional images were additionally smoothed with a 6-mm full-width at half maximum isotropic Gaussian kernel.

Normalization

The functional images were spatially normalized to MNI space.

Normalization template

MNI

Noise and artifact removal

First, we applied field mapping distortion correction to the functional volumes to correct for geometric distortions in EPI caused by magnetic field inhomogeneity (with the FieldMap toolbox). The functional images of all runs were realigned and the individual structural T1 image were co-registered to the mean functional image generated during realignment. The functional images were spatially normalized to MNI space. For the univariate analysis the functional images were additionally smoothed with a 6-mm full-width at half maximum isotropic Gaussian kernel.

Volume censoring | none

Statistical modeling & inference

Model type and settings

Data of all four functional runs were analysed using the general linear model (GLM) with a 128 s high pass filter. We applied SPM’s alternative pre-whitening method to account for autocorrelation, FAST, which has been suggested to perform better than SPM’s default. Raw motion parameters (three translations and three rotations), their derivatives, squared derivatives, as well as the average time-course of the left and right ventricles were included as nuisance covariates.

Effect(s) tested

For the localizer: On the second level, we computed the t-contrast ‘face images > scene images’ to localize face-sensitive regions.
In addition, to investigate the effect of face expectation strength on the activation during face presentation, we set up a GLM with a parametric modulator on the presented faces. Specifically, we tested for 1) an increased signal in response to presented faces that were less expected by means of a negative linear parametric modulator, 2) an increased signal in response to presented faces that were more expected with a positive linear parametric modulator, and 3) a u-shaped univariate response reflecting increased activity for highly expected and unexpected with a quadratic parametric modulator. On the second level, we tested for the linear modulator whether it was larger or smaller than 0, respectively, and for the quadratic modulator whether it was larger than 0.

Specify type of analysis: ☐ Whole brain ☐ ROI-based ☒ Both

Anatomical location(s)

We defined our regions of interest (ROIs) based on the functional localizer run before participants learned the associations between face and scene images.

Statistic type for inference
(See Eklund et al. 2016)

The univariate analysis was performed in the whole brain.
The RSA analysis was perfomred within pre-defined ROIs.

Correction

p<0.001 inducing threshold, cluster FWE-corrected

Models & analysis

n/a	Involved in the study
☒	☐ Functional and/or effective connectivity
☒	☐ Graph analysis
☒	☐ Multivariate modeling or predictive analysis
